# The Effectiveness of Early Childhood Caries Treatment with MI Varnish Fluor in Obese Subjects: A Study from Vietnam

**DOI:** 10.3390/children8121151

**Published:** 2021-12-07

**Authors:** Ha Van Hung, Vo Truong Nhu Ngoc, Dinh-Toi Chu

**Affiliations:** 1 School of Odonto Stomatology, Hanoi Medical University, Hanoi 100000, Vietnam; drhahungtb@gmail.com; 2 Center for Biomedicine and Community Health, International School, Vietnam National University, Hanoi 100000, Vietnam

**Keywords:** childhood obesity, early childhood caries, MI Varnish Fluor

## Abstract

***Objective***: We conducted this work to evaluate the effectiveness of treatment for early childhood caries (ECC) using MI Varnish Fluor in obese children aged from 36 to 71 months. ***Methods***: This study was conducted on 300 carious teeth of obese children and normal-weight children in Hanoi, Vietnam, over the period 2019–2020. Diagnodent KaVo 2190 laser equipment was used to diagnose ECC. The children in each group were selected on the basis of similarities in age, gender, and study location, and the teeth in the two groups were selected on the basis of similarities in damage level and jaw position. ECC treatment was performed once a week for four consecutive weeks with MI Varnish Fluor. The child, the child’s family, and the child’s teacher were consulted on diet and oral hygiene during the treatment. Children were examined and monitored throughout the treatment period. Children were re-examined after 3 and 6 months from the start time of treatment. The Mann–Whitney U test and Kruskal–Wallis tests were used, with statistical significance indicated at *p* < 0.05. ***Results****:* After six months of treatment with MI Varnish Fluor, the number of cases of code 0 damage recovery (D0) increased in both groups. The result showed that MI Varnish fluor was effective in ECC treatment. D0 damage recovery rates of 79.3% in obese children and 62.7% in normal-weight children were observed after six months of treatment, but there was no statistically significant difference between the two groups. Furthermore, there was no statistically significant difference between the two groups according to age, tooth position, or tooth surface position in D0 damage recovery. ***Conclusions****:* MI Varnish Fluor was effective in ECC treatment, with D0 damage recovery rates of 79.3% in obese children and 62.7% in normal-weight children after six months of treatment.

## 1. Introduction

ECC is a community issue that affects children around the world [1]. According to the American Academy of Pediatric Dentistry (AAPD), ECC is one or more instances of tooth damage, tooth loss due to decay, or filled cavities on any milk tooth in babies between birth and 71 months of age [2]. Compared with other age groups, the rate of early decay is around 60–90% and is the highest in Asia and Latin America [3]. In Vietnam, according to the study results of Nguyen YHT et al., the rate of ECC in children aged 3–5 years is 79.1% [4], and according to Do MH et al., this rate in 4-year-old children is 92% [5]. Moreover, tooth decay has not received sufficient attention, especially in preschool-age children. Khanh L.N et al. showed that the proportion of preschool-aged children with tooth decay was up to 95.4% [6]. Untreated ECC progresses rapidly, destroying the crown and causing pain. Its consequences are not only harmful to children’s health but also costly. Therefore, this is a challenge for all countries around the world, even developed ones [1].

On the other hand, the relationship between obesity and tooth decay in children has been shown in previous studies [7]. However, tooth decay and obesity are both multi-factor diseases, and their association is quite complex [8]. Factors affecting tooth decay in obese children such as eating habits and oral hygiene habits have been proven [9,10]. Thus, there is a hypothesis that there is a difference in ECC treatment efficacy between obese and non-obese children after the treatment time and the re-examination time. However, due to medical ethics, we cannot conduct research on a treated obese group versus an untreated obese group, or a group receiving treatment and intervention in eating and living habits with a group receiving treatment and no intervention in eating and living habits. Therefore, we conducted this study to check if there was any difference in the treatment effect of fluoride varnish for caries on obese children compared to normal weight children.

The detection, prevention, and treatment of ECC have been some of the priorities of dentistry, especially in preschool children. Varnish Fluor (NaF5% and casein phosphopeptide–amorphous calcium phosphate (CPP-ACP)) was developed in the late 1960s [1], and it is effective in the prevention and treatment of white spot lesions of enamel. According to the International Caries Classification and Management System (ICCMS) classification, ECC in preschool children is a discoloration or white spot lesion of the enamel [11]. Prevention and treatment of ECC with Varnish Fluor has been proved as a professional, effective, safe, and convenient method that is well-tolerated in children [1,12,13,14].

Luu VT et al. conducted a study to evaluate the effectiveness of tooth decay treatment with Varnish Fluor in 3-year-old children in Hanoi, Vietnam in 2020 [15]. They showed that the rate of tooth decay in 3-year-old children was 78.6%, of which ECC accounted for 64.5%. After 18 months of treatment, the ECC rate (D1, D2) reduced from 100% to 66.1%. From that result, scientific evidence was provided that Varnish Fluor is influential in preventing and treating ECC in 3-year-old children in Vietnam. However, using Varnish Fluor for ECC treatment in Vietnam is not expected at present. It has also not been systematically coordinated with other methods, so optimal effects have not been achieved. This is partly due to the lack of in-depth studies to lay the foundation for the application of Varnish Fluor in clinical practice. In the world as well as in Vietnam, there has been research on the effectiveness of ECC treatment with Varnish Fluor in normal children. However, there are few reports of this issue in obese children, especially in obese preschool-aged children. Furthermore, eating habits and nutrition have been implicated as risk factors for obesity and ECC. Thus, our study was conducted to evaluate the effectiveness of ECC treatment with MI Varnish Fluor in obese children aged from 36 to 71 months in Hanoi, Vietnam, during the period 2019–2020.

## 2. Research Subjects and Methods

### 2.1. Research Subjects

Group classification was performed based on body mass index (BMI). Based on Barlow and Expert’s proposed cut-off points, Al Herbish classified BMI by age and gender: <5th percentile, underweight; 5th–84th percentile, normal weight; 85th–94th percentile, overweight; and >95th percentile, obese [16].

In total, 150 carious teeth of obese children and 150 carious teeth of normal-weight children were diagnosed using The Diagnodent KaVo 2190 laser equipment and were classified based on the ICCMS classification. Children were selected with the voluntary consent of their parents or guardians.

### 2.2. Selection Criteria

Obese and normal-weight children aged from 36 to 71 months were selected [9]. Teeth were diagnosed as ECC according to the ICCMS classification [11,12,13]: First or distinct visual changes in enamel seen as a white spot lesion and/or brown carious discoloration, not consistent with the clinical appearance of sound enamel and which show no evidence of surface breakdown or underlying dentine shadowing. Their parents or guardians gave voluntary consent via a written form.

### 2.3. Exclusion Criteria

Subjects were free to withdraw at any stage of the research. The following were excluded from the study: children who did not cooperate during the examination and treatment; children with psychological diseases or maxillofacial diseases; and children who were allergic to Fluor, were taking fluoride antagonists, or had taken fluoride within the previous six months.

### 2.4. Methods

- *Time and place of research*

+ Time: From March 2019 to December 2019.

+ Place of study: Selected kindergartens in four suburban districts of Hanoi, Vietnam (Phu Xuyen district, My Duc district, Hoai Duc district, and Thuong Tin district).

- *Research design*: A clinical trial.

- *Sample size:* Sampling of all 300 teeth diagnosed as ECC, including 150 teeth in obese children and 150 in normal-weight children.

- *Sample selection:*

For this study, we used the convenience sampling method. The children in the obese and normal-weight groups were selected on the basis of similarities in age, gender, and study location. The teeth in the two groups were selected on the basis of similarities in damage level and jaw position. Sampling was stopped when each group had 150 teeth with ECC.

- *Data collection:*

Clinical examination was carried out with equipment such as a dental examination mirror, smartphones to monitor and save images, and the Diagnodent KaVo 2190 laser (Germany). The Diagnodent KaVo 2190 laser was used according to the manufacturer’s instructions, and the index symbol was Di. Classification of ECC was performed according to The International Caries Detection and Assessment System II (ICDAS II) [17]. This system has six different classification codes: 0, sound; 1, first visual change in enamel; 2, distinct visual change in enamel; 3, localized enamel breakdown; 4, underlying dentine shadow; 5, distinct cavity with visible dentine; and 6, extensive cavity with visible dentine. This was checked with the Dianogdent [18] KaVo 2190 fluorescent laser equipment. This device can detect back-scattered fluorescence from teeth through a sensor that emits 655 nm monochromatic light [19]. The DD scores are divided into three levels which range from 0 to 99. The diagnostic criteria for ECC were divided into 4 levels: no caries (D0) with laser Di index from 0 to 13; ECC level 1 (D1) with laser Di index from 14 to 20; ECC level 2 (D2) with laser Di index from 21 to 29; and dental caries level 3 (D3) with laser Di index ≥30. Two prosthodontists and five dental nurses performed the examinations and data collection.

- *Treatment process*

The process began after cleaning the child’s teeth with a soft brush and water. Teeth diagnosed with ECC were isolated with medical cotton, then a thin layer of Varnish Fluor (GC Corporation, Itabashi-Ku, Tokyo, Japan) was applied to the surface and dried in four minutes. Children were not allowed to eat or drink for the first 2 h; they could snack after 4 h and brush their teeth after 24 h. The treatment was applied four times, once a week. The child, the child’s family, and the child’s teacher were consulted on diet and oral hygiene during the treatment. Children were examined and monitored throughout the treatment period. Children were re-examined after 3 and 6 months from the start time of treatment.

### 2.5. Statistical Analysis

The data were entered into Epidata and Stata 14.2 software was used for data analysis. The Mann–Whitney U test and Kruskal–Wallis tests were used, with statistical significance indicated at *p* < 0.05.

## 3. Results

In total, 137 children were selected, of which 66 were obese and 71 were of normal weight, accounting for 51.82% and 78.18%, respectively. Furthermore, 60% of the total were female. Five-year-old children accounted for the highest percentage (56.20%), followed by four-year-old children (29.20%) and, finally, three-year-old children (14.60%) (Table 1).

In the obese group, ECC accounted for 26.65%, including 65 instances of damage at the D1 level (11.55%) and 85 instances of damage at the D2 level (15.10%). There were 416 instances of damage at the D3 level in the obese group, accounting for 73.55%. In the normal-weight group, the percentage of ECC was 31.64%, including 65 instances of D1 damage (13.71%) and 85 instances of D2 damage (17.93%). Instances of D3 damage in the normal-weight group accounted for 68.36%. The two groups presented similarities in damage with age: 3-year-olds presented 28 instances of damage with ECC, including 13 D1 and 15 D2; 4-year-olds presented 50 instances of damage with ECC, including 20 D1 and 30 D2; and 5-year-olds presented 72 instances of damage with ECC, including 32 D1 and 40 D2. Similarly, the positions of teeth were selected to be equal between the two groups. The number of instances of damage was highest in the molars and lowest in the canines (Table 2).

After six months of treatment with MI Varnish Fluor, the obese male children showed a higher number of instances of D0 recovered damage (81 instances of damage) than the normal-weight male children (48 instances of damage). The obese female children showed a lower number of instances of D0 recovered damage (38 instances of damage) than the normal-weight female children (46 instances of damage). Even so, all of the above differences were not statistically significant. On the other hand, we found that the difference was statistically significant in D1 damage between the obese female children and the normal-weight female children. The normal-weight female children presented a higher number of instances of D1 damage. There was no statistically significant difference in D2 and D3 damage between the two groups in both sexes (Table 3).

The number of instances of D0 recovered damage in obese children was higher than that in normal-weight children in all age groups, but these differences were not statistically significant. On the other hand, 5-year-old children presented the highest number of instances of D0 recovered damage in both groups, followed by 4-year-old children, with the lowest in 3-year-old children. These differences were also not statistically significant (Table 4).

The D0 recovered damage was different among the tooth positions after six months of treatment. In the obese group, the molars in the upper jaw had the highest number of D0 cases, and the incisors in the lower jaw had the lowest. In the normal-weight group, the number of D0 cases was highest in molars in the upper jaw and lowest in molars in the lower jaw. All of these differences were not statistically significant (Table 5).

The number of teeth with ECC (D1 and D2) in both groups gradually decreased during the treatment time, and the number of instances of D0 recovered damage steadily increased. The difference in D0 recovered damage over the time points was statistically significant (*p* < 0.001). In obese children, the effectiveness of treatment after six months was 79.3% (119/150), at which point 17 instances of D2 damage and 6 instances of D3 damage remained. In normal-weight children, treatment effectiveness was lower than that in the obese children, with 62.7% recovery after six months (119/150), presenting 25 instances of D2 damages and 11 instances of D3 damage that were not cured. After six months of treatment with Varnish Fluor, we did not see a statistically significant difference in the effectiveness of treatment between obese and normal-weight children (Table 6).

## 4. Discussion

The obese and normal-weight children in this study received the same treatment and 6-month follow-up. Each child, the child’s family, and the child’s teacher were consulted on a diet to reduce the sugar in their food and drink and change the children’s snacking habits. In particular, we had a guide for their parents on tooth brushing with fluoride toothpaste. The guide proposed the dosage, brushing time, and frequency as recommended by the Academy of Pediatric Dentistry. After six months, the children’s parents continued to receive counseling, and the treatment continued for children with severe progression.

After one month, three months, and six months of follow-up, the effectiveness of the treatment gradually increased. The effectiveness was 79.3% and 64.7% in obese and normal-weight children, respectively. These rates are higher than those in Poulami Mishra’s study, with 5–63% recovered damage after treatment with Varnish Fluor [1], and higher than that in Rao’s study, which found 69.7% recovered damage in children aged 2–3 years [16]. D Duangthip et al. conducted a randomized trial in 304 children aged 3–4 years. In that study, 1670 instances of damage were randomized into three groups: Group 1, SDF (Silver Diamine fluoride) 30% applied every 12 months; Group 2, SDF 30% applied three times, once per week; and Group 3, Varnish Fluor NaF 5% applied three times, once per week. After 18 months of treatment, the rate of inactive damage was 40% in Group 1, 35% in Group 2, and 27% in Group 3, with *p* < 0.001 [20]. D Duangthip et al., 2018, also conducted clinical trials on 309 children aged 3–4 years, in which 1877 instances of damage were divided into three groups, similar to those in previous studies. After three months of treatment, the rate of inactive damage at level 5 or level 6 according to the ICDSA classification was 48% in Group 1, 33% in Group 2, and 34% in Group 3, with *p* < 0.001 [21]. Furthermore, Sarangapani Radha’s study aimed to monitor the effectiveness of treatment for 3 and 12 months. Sarangapani Radha reported 90% and 100% recovered damage after treatment [22]. The age range from 3 to 5 years in our research subjects can partly explain this difference. At this age, children have started showing awareness and cooperation in the process of treatment. Furthermore, while other studies only selected level 2 damage in the ECC stage, our research subjects were selected with both level 1 and level 2 ECC damage. Moreover, in our study, we consulted on diet and oral hygiene with the child’s family and the child’s teacher, contributing to the higher effectiveness of treatment.

The recovery rates in the normal-weight and obese groups were different. After the treatment, 17 instances of D2 damage and 6 instances of D3 damage remained in the obese group, while 25 instances of D2 damage and 11 instances of D3 damage were not cured in the normal-weight group. However, all these differences were not statistically significant. Previous scientific evidence has shown that tooth decay in obese children is related to excessive sugar intake and the children’s eating habits. However, the results of our study showed that if children were treated with Varnish Fluor in combination with changing eating habits, the effectiveness of ECC treatment was favorable. Moreover, there have not been many types of research on the efficacy of ECC treatment with Varnish Fluor in obese children. We hope that longitudinal follow-up studies with a longer duration will be carried out in the future and shed further light on this issue.

Regarding the treatment effect for the different tooth positions, the molars in the upper jaw had the highest number of D0 in both groups. The incisors in the lower jaw and molars in the lower jaw had the lowest numbers in the obese and normal-weight groups. These results were similar to those of a meta-analysis that showed that the upper jaw’s molars had a better treatment response than others [23]. This result can be explained by the fact that the position of the molars made it easier for the dentist to apply Varnish Fluor. Moreover, the molars could retain the drug longer on the surface, contributing to better treatment efficiency.

Our research had some limitations. Non-random sampling reduced the representativeness of our study results. This study did not evaluate the examiner’s calibration during treatment. Furthermore, for this study, we mainly used foreign references due to the lack of research in Vietnam, so there was little similarity with Vietnamese children. Moreover, our study has yet to compare other tooth decay treatments with MI Varnish Fluor.

## 5. Conclusions

After six months of treatment with MI Varnish Fluor, the D0 damage recovery rates increased in both groups. MI Varnish Fluor was effective in ECC treatment, with D0 damage recovery rates of 79.3% in obese children and 62.7% in normal-weight children after six months of treatment; there was no statistically significant difference between the two groups. There was also no statistically significant difference between the two groups according to age, tooth position, or tooth surface position in D0 damage recovery.

## Figures and Tables

**Table 1:** Characteristics of research subjects (*n* = 137).

Characteristics		*n*	%
**Obesity**	Yes	66	48.18
	No	71	51.82
**Gender**	Male	53	38.69
	Female	84	61.31
**Age**	3 years old	20	14.60
	4 years old	40	29.20
	5 years old	77	56.20

**Table 2:** Dental caries characteristics of research subjects.

	Obesity						Normal Weight					
	D1		D2		D3		D1		D2		D3	
	*n*	%	*N*	%	*n*	%	*n*	%	*n*	%	*n*	%
**Total**	65	11.55	85	15.10	413	73.35	65	13.71	85	17.93	324	68.36
**Gender**												
Male	43	7.64	59	10.48	275	48.85	25	5.27	51	10.76	201	42.41
Female	22	3.91	26	4.62	138	24.50	40	8.44	34	7.17	123	25.95
**Age**												
3 years old	13	2.31	15	2.66	70	12.43	13	2.74	15	3.16	57	12.03
4 years old	20	3.55	30	5.33	147	26.11	20	4.22	30	6.33	103	21.73
5 years old	32	5.69	40	7.11	196	34.81	32	6.75	40	8.44	164	34.60
**Tooth Position**												
Molars in the upper jaw	17	3.02	16	2.84	106	18.83	17	3.59	16	3.38	80	16.88
Molars in the lower jaw	15	2.66	16	2.84	138	24.51	15	3.16	16	3.38	92	19.41
Incisors in the upper jaw	15	2.66	20	3.55	98	17.41	15	3.16	20	4.22	94	19.83
Incisors in the lower jaw	4	0.71	6	1.07	22	3.91	4	0.84	6	1.27	21	4.43
Canines in the upper jaw	10	1.78	16	2.84	29	5.10	10	2.11	16	3.38	21	4.43
Canines in the lower jaw	4	0.71	11	1.95	20	3.55	4	0.84	11	2.32	16	3.38

**Table 3:** Effectiveness of treatment after 6 months by gender.

	Obesity			Normal Weight			*p*-Value (Male)	*p*-Value (Female)	*p*-Value (Total)
	Male	Female	Total	Male	Female	Total			
**D0**	81	38	119	48	46	94	0.142	0.932	0.232
**D1**	6	2	8	9	11	20	0.310	0.031 ^#^	0.026 ^#^
**D2**	13	4	17	12	13	25	0.793	0.083	0.188
**D3**	4	2	6	5	6	11	0.513	0.128	0.682

Note: The Mann–Whitney U test was used to compare the mean values of D0, D1, D2, and D3 damage between two groups, ^#^ *p* < 0.05.

**Table 4:** Effectiveness of treatment after 6 months by age.

Age	Obesity				Normal Weight				*p*-Value D0 **
	D0	D1	D2	D3	D0	D1	D2	D3	
**3 years old**	12	2	2	0	10	5	4	3	0.089
**4 years old**	35	4	7	2	26	7	6	2	0.433
**5 years old**	72	2	8	4	58	8	15	6	0.202
*p*-Value *	0.340	0.131	0.762	0.751	0.104	0.681	0.967	0.139	

Note: * The Kruskal–Wallis test was used to compare the mean values of D0, D1, D2, and D3 damage by age and ** the Mann–Whitney U test was used to compare the mean value of D0 between two groups.

**Table 5:** Effectiveness of treatment after 6 months by tooth position.

Tooth Position	Obesity				Normal Weight				*p*-Value (D0)
	D0	D1	D2	D3	D0	D1	D2	D3	
**Molars in the upper jaw**	30	1	2	1	22	4	6	3	0.338
**Molars in the lower jaw**	21	2	6	3	6	2	3	1	0.888
**Incisors in the upper jaw**	27	3	3	1	22	5	5	2	0.839
**Incisors in the lower jaw**	8	0	2	0	21	4	5	2	0.644
**Canines in the upper jaw**	20	2	3	1	14	4	5	3	0.265
**Canines in the lower jaw**	13	0	1	0	9	1	1	0	0.458

Note: The Mann–Whitney U test was used to compare the mean value of D0 damage between two groups.

**Table 6:** Effectiveness of treatment over the examined time points.

	Obesity				Normal Weight				*p*-Value (D0 **)
	D0	D1	D2	D3	D0	D1	D2	D3	
**Before treatment**	0	65	85	0	0	65	85	0	-
**1 month**	22	55	73	0	17	57	76	0	0.647
**3 months**	68	35	47	0	66	42	42	0	0.383
**6 months**	119	8	17	6	94	20	25	11	0.232
*p*-Value *	<0.001	<0.001	<0.001	-	<0.001	<0.001	<0.001	-	

Note: * The Kruskal–Wallis test was used to compare the mean values of D0, D1, D2, and D3 damage over the examined time points, and ** the Mann–Whitney U test was used to compare the mean value of D0 between two groups.

## Data Availability

Data are contained within the article.
